# Standoff Distance in Ultrasonic Pulsating Water Jet

**DOI:** 10.3390/ma14010088

**Published:** 2020-12-27

**Authors:** Madhulika Srivastava, Akash Nag, Somnath Chattopadhyaya, Sergej Hloch

**Affiliations:** 1 Department of Mechanical Engineering, Amrita School of Engineering, Amrita Vishwa Vidyapeetham, Chennai 601103, India; s_madhulika@ch.amrita.edu; 2 Department of Mechanical Engineering, Indian Institute of Technology (Indian School of Mines), Dhanbad 826004, India; akashnag1992@gmail.com (A.N.); somnathchattopadhyaya@iitism.ac.in (S.C.); 3 Faculty of Manufacturing Technologies, Technical University of Kosice, Prešov, Bayerova 1, 080 01 Presov, Slovakia; 4 Institute of Geonics of the Czech Academy of Sciences, Studentska 1768, 708 00 Ostrava-Poruba, Czech Republic

**Keywords:** pulsating water jet, stainless steel, traverse speed, disintegration depth

## Abstract

The water hammer effect is the basis of technologies which is artificially responsible for the decay of continuous jets. A recently developed technique enhances the pressure fluctuations using an acoustic chamber, leading to enhanced erosion effects for various water volume flow rates. The optimum standoff distance for an ultrasonic enhanced water jet is not appropriately estimated using an inclined trajectory. The objective of this study is to comprehend the true nature of the interaction of the standoff distance following the stair trajectory and traverse speed of the nozzle on the erosion depth. Additionally, it also critically compares the new method (staircase trajectory) that obeys the variation in frequency of the impingements for defined volume flow rates with the inclined trajectory. In this study, at constant pressure (*p* = 70 MPa), the role of impingement distribution with the variation of traverse speed (*v* = 5–35 mm/s) along the centerline of the footprint was investigated. The maximum erosion depth corresponding to each traverse speed is observed at approximately same standoff distance (65 ± 5 mm) and decreases with the increment in traverse speed (*h* = 1042 and 47 µm at *v* = 5 and 35 mm/s, respectively). The results are attributed to the variation in the number of impingements per unit length. The surface and morphology analysis of the cross-section using SEM manifested the presence of erosion characteristics (micro-cracks, cavities, voids, and upheaved surface). By varying the water cluster, different impingement densities can be achieved that are suitable for technological operations such as surface peening, material disintegration, or surface roughening.

## 1. Introduction

The concept of hydrodynamic erosion has been studied for improving our understanding of the mechanisms involved during the interaction of a liquid with a solid under different conditions. These interactions can be disadvantageous for engineering applications such as jet engines, turbine blades, rain erosion in wind turbines, aircrafts, and helicopters [1], but are advantageous for applications such as high-speed water jet cutting [2], peening, and surface treatments. The impingement of a liquid jet or droplet on a surface (solid) causes deformations due to the initial normal impact of the jet, which is known as the water hammer effect, followed by stress wave propagation and shear force induced during lateral jetting [3]. In ductile materials, erosion occurs due to plastic deformation, where the material undergoes a large amount of plastic strain before fracture. However, in brittle materials, the erosion occurs due to crack propagation and interaction of the initiated crack with other cracks generated in the surface and sub-surface of the material [4].

In continuous water jet (CWJ) technology, stagnation pressure prevails over impact pressure, which acts during the initial contact of the jet with the solid surface. However, the impact pressure (*p_i_*) is higher compared to the stagnation pressure (*p_s_*) for the same velocity of the jet (Equations (1) and (2)), inducing higher compressive stresses into the material during the interaction.

(1) $$p_{i}=\frac{v_{w}\rho_{1}c_{1}\rho_{2}c_{2}}{\rho_{1}c_{1}+\rho_{2}c_{2}}$$

(2) $$p_{s}=\frac{1}{2}\rho_{1}{v_{w}}^{2}$$

where $\rho_{1}$ and $\rho_{2}$ are densities and $c_{1}$ and $c_{2}$ are shock wave velocities in liquid and solid media, respectively, and $v_{w}$ is jet velocity. Therefore, research is being conducted by exploiting the conversion of continuous jets into discrete water clusters in order to enhance surface properties and material disintegration efficiency using low technological input (supply pressure (*p*) < 100 MPa). Several methods for modifying a continuous jet into discrete water clusters are achieved through rotating disks, self-resonating nozzles, Helmholtz resonators, and ultrasonic needles. However, some limitations such as the limited-service life of the mechanical components, complex design, and reduction in the erosion with minor changes in the physical properties of the jet are observed with above methods. These limitations were overcome by a prototype designed by Foldyna [5], which uses an ultrasonic sonotrode oscillating inside an acoustic chamber for efficient propagation of shock waves. In the pulsating water jet (PWJ), the ultrasonic generator excites the piezoelectric ceramics attached to the sonotrode, which, in turn, oscillates with a frequency (*f*) of 20 kHz. The water flow interacts with the sonotrode inside a pressurized acoustic chamber. The vibration of the solid sonotrode induces pressure fluctuations in the form of standing waves inside the acoustic chamber. Appropriate setting of the chamber length tunes the solid sonotrode to resonate with the water sonotrode formed inside the chamber to increase the efficiency of the ultrasonic power. The amplitude of the pressure fluctuations in the form of standing waves increases toward the nozzle exit due to its converging shape. At the nozzle exit, the pressure fluctuation transforms into velocity fluctuation, resulting in variation in the axial velocity of the jet along the axis. This variable jet velocity forces the continuous jet of the water to break into discrete bunches of water droplets, inducing impact pressure onto the material.

The erosion behavior of various materials depends on the PWJ process parameters (Table 1). The main process parameters associated with PWJ technology are supply pressure (*p*), traverse speed of the nozzle (*v*), standoff distance (*z*), acoustic chamber length (*lc*), frequency of ultrasonic transducer (*f*), nozzle diameter (*d*), and nozzle geometry. To investigate the response of erosion to the process, different studies have been performed. During the initial investigation [6], aluminum alloy was treated to determine the effect of various parameters (operating pressure and amplitude of vibration) on the erosion. The samples were treated at pressures of 20, 30, and 40 MPa with a traverse speed of 0.1 m/min, using a fan jet nozzle of diameter 2.05 mm, varying standoff distance from 30 to 90 mm, with vibration amplitudes of 3, 5, and 7 µm. The response of the jet was observed in terms of the volume removal and surface roughness. The findings showed that the optimum standoff distance increases with the increase in the operating pressure, as the breakup length of the jet increases proportional to the jet velocity. The volume removal results showed the existence of three regions of PWJ action on the surface: no erosion, erosion by impact of developed water waves, and typical droplet erosion. A stainless steel surface treated at operating pressures ranging between 10 and 30 MPa, with a standoff distance of 40 mm for 10 MPa and 50 mm for 20 and 30 MPa, also showed the existence of three regions of PWJ action on the surface [7]. The behavior of the surface characteristics during erosion was also observed by means of surface roughness profiles (*Ra* and *Rz*) for a number of PWJ conditions.

The erosion phenomenon was also studied for copper alloys (bronze and brass) [8] in terms of surface topography, morphology, and anisotropy, created by a PWJ with a frequency of 20.38 kHz. The PWJ flowed from a flat nozzle with a diameter of 2 mm and theoretical speed of 254.81 m/s, acting on untreated surfaces. Surface quality was determined by surface roughness profile parameters (*Ra* and *Rz*). The authors observed that the material removal rate and the related groove depth, *h* (mm), increased with increasing number of passes (*n*). The surface integrity of copper and brass was examined in terms of changes in surface and subsurface layers [9]. The erosion traces created using the PWJ were observed to depend on the material’s structure and its mechanical properties. A parametric study (variation in traverse speed, nozzle diameter, and hydraulic power) conducted through full factorial analysis of experiments (3^3^) on the brass solid flat surfaces showed the erosion effects in terms of mass material removal [10]. The variation in the nozzle diameter from 1.30 to 1.60 mm did not have significant effects on the disintegration. The increase in hydraulic power affected the disintegration with increasing mass material removal.

An initial test of mechanical properties (microhardness and nanoindentation) showed that PWJ can be used for easy removal of the cemented femoral stem from the femoral channel in revision surgery [11]. The comparison with continuous and pulsating water jets for disintegrating bone cement demonstrated the effectiveness of water jet technology for reimplantation of cemented endoprostheses, without heat generation or damage to the surrounding tissue [12]. The disintegration of the interface between the cemented femoral stem and trabecular bone tissue, created by bone cement, was also examined at pressures ranging between 8 and 20 MPa using different nozzle geometries (flat and circular) with other laboratory conditions, as listed in Table 1. The traces generated by the pulsating water jet using the circular nozzle resulted in deeper grooves. The disintegration of the interface between the cemented femoral stem and trabecular bone tissue, created by bone cement, for the application in revision arthroplasty was also investigated [13]. Six types of commercial bone cements were disintegrated at pressures in the range 8–20 MPa, as described in Table 1. The mechanical properties were evaluated by nanoindentation and the material removal volume, while the created trace depths were measured using a MicroProf FRT non-contact optical profilometer. An initial investigation proved that this technology is quick and safe for bone disintegration in arthroplasty revision. During the study of the effect of PWJ decay for bone cement removal [14], with increasing standoff distance (*z* = 4 to 20 mm), the depth of the groove initially increased, followed by a decrease (*h* = 565, 615, and 418 μm). The variation in the acoustic chamber length up to 22 mm increased the disintegration depth of the bone cement. With the decrease in traverse speed (*v* = 2 to 0.5 mm/s), groove depth increased to 527 and 599 μm, respectively; however, a reverse effect of the depth was observed with decreasing supply pressure.

The effect of nozzle geometry (circular and flat) during the peening of austenitic stainless steel surfaces using a PWJ improved the surface residual stresses and surface and subsurface hardness [15]. The variation in parameters (supply pressure, traverse speed, and standoff distance) during peening showed that a lower pressure of 40 MPa, a traverse speed of 5 mm/s, and an optimum standoff distance of 31 mm produced the maximum increase in the residual stress (540 MPa) and micro-hardness (570 HV) [16]. A similar effect was observed in AISI 304 welded joint surfaces treated at pressures of 20, 40, and 60 MPa [17]. For different combinations of traverse speeds and standoff distances, an improvement in surface residual stress and subsurface micro-hardness was observed. The results obtained showed the potential for the use of the technology for peening applications.

The hydrodynamic ductile erosion of aluminum [18] treated along an inclined trajectory showed distinct erosion regimes in terms of surface features. The standoff distance ranging from 4 to 90 mm showed the presence of micro-voids, followed by crack initiation and propagation and, finally, material removal with upheaving characteristics.

The acoustic chamber was found to be the most important parameter as it controls the variable axial jet speeds [19]. The relationship between the acoustic chamber length and standoff distance was studied on AW 6060 aluminum alloy. The maximum depth of the eroded trace was hyperbolic in shape with a maximum depth reached with the increase in standoff distance (5–101 mm) along a staircase trajectory. Additionally, at a higher operating pressure (*p* = 40 MPa), deeper groves were obtained.

A study of process parameters during granite erosion [20] showed that the traverse speed with a combination of pressure and standoff distance decreases the width and volume of erosion; however, no particular trend was observed for the depth of the cut. The frequency change (20 and 40 kHz) analysis during sandstone erosion [21] showed that the maximum volume of erosion occurred with the maximum depth and minimum width which is obtained at a higher frequency level of 40 kHz due to the higher number of impacts at higher frequencies.

**Table 1:** Results of recent studies dealing with the evaluation of pulsating water jet (PWJ) interactions.

Material	Parameters								
	Pressure (MPa)	Frequency (kHz)	Nozzle Type	Nozzle Diameter (mm)	Acoustic Chamber Length (mm)	Standoff Distance (mm)	Traverse Speed (mm/min)	No. of Passes	Study
Aluminum	20		Flat	2.05–15°	3, 5, 7	20–90	0, 1	1	[6]
	30								
	40								
AISI 316Ti	10	21.25	Circular	1.6	7	40	0	1	[7]
	20					50			
	30					50			
Brass, Bronze	40	20.38	Flat	2	-	55	2, 4	2/4	[8]
CW004A CW614N	38	20.29	Circular	1.6	-	48	0.75	1	[9]
Brass		20.31	Circular	1.067	-	35	0.50	1	[10]
	39			1.321			0.75		
				1.600			1.00		
G bone cement, Palacos R + G^®^	8–16	41.90	Flat	0.8–10°	n.a.	2	1	1	[11]
						4			
PMMA	8–20	41.90	Circular	0.7		4	1	1	[12] [13]
	8–12	41.90	Flat	0.8–10°		2	1	1	
	40	n.a.	Circular	0.1	n.a.	2–3	1	1	
	120	n.a.	Circular	0.1	n.a.	2–3	1	1	
Palacos R + G	5, 6, 7, 8, 9, 10	21	Circular	0.3	0–22	2–20	0.5, 1, 2	1	[14]
AISI 304	20	20	Flat	1.0–10°	16	30	0.25, 1.0, 1.5, 2.5	1	[15]
			Circular	1.9		45			
AISI 304 Welded	20	20	Circular	1.9	16	45	1, 2	1	[17]
	40					70	3, 4		
	60					100	5, 6		
AW-6060	100	20	Circular	0.9	6	5–90	10	1	[18]
						25			
						75			
						125			
		0	Circular	0.9	6	25			
AW-6060	30 ± 1	20.40	Circular	0.6	5–22	5–101	5	1	[19]
	40 ± 1								
Granite	20	20	Circular	1.9	16	45	5, 4, 3, 2, 1	1	[20]
	40					70	5, 6, 7, 8, 9		
	60					100	9, 11, 15, 17, 19		
Sandstone	20	20, 40	Circular	1.6	-	20	125, 150, 175, 200	1	[21]
	30								
	40								
EN AW 5083 H111	20	20.09	Flat	2	7	55	0.5	1	[22]
							0.75		
							1.0		
							2.0		
							4.0		
Concrete Composite	40	20	Circular	1,9	7	35	50–160	1	[23]
	20								
AISI 304	40–100	20	Circular	1.19	22	5–101	5	1	[24]
Sandstone	20	20, 40	Circular	1.9	-	5–55	0.25	1	[25]
							0.5		
							0.75		
							1		

Two important factors affecting the jet decay and erosion rate of the PWJ are the frequency of the sonotrode and the acoustic chamber length. The frequency must be designed to generate a further reduction in amplitude in the PWJ for the decay of the continuous water jet. Research has shown that the amplitude requires a Strouhal number (*St*) of 0.3, which can be determined using:

- Relaxation: The time between the impacts of the water clusters on the material should be long enough to allow full relaxation of the energy of the previous cluster.
- Attenuation: The time between the impacts of the clusters on the material should always be long to avoid attenuation of the energy of the impacting droplets on the material, affecting the presence of liquid from the previous cluster.
- Aerodynamics: Immediately after the formation of a cluster of liquid, the air drag force begins to act on this cluster, decaying it into small droplets. The effects of aerodynamic drag are reduced if each cluster tends to have a protected cluster following it [26].

The previous studies [8,10,19,25] that have been reported in Table 1 used stationary or inclined trajectory movement of the PWJ head to determine the optimal standoff distance corresponding to the maximum depth of disintegration. However, in the present article, a new methodology in the form of staircase trajectory movement has been used to include the vertical component of the traverse speed (*v_y_* = *v*·sin*α* mm/s; *α* is the angle of inclination of the inclined path), which was omitted in the previous studies.

Therefore, we followed the study [24] that proposed and used staircase trajectory methodology for estimation of standoff distance enriched by observation of the selected pressure (*p* = 70 MPa) for varying traverse speeds (*v* = 5–35 mm/s). Our main aim was to understand the interaction of increasing standoff distance (*z* = 5 to 101 mm) and traverse speed on the disintegrating depth of AISI 304 material, as well as to estimate the optimal jet decay length of a PWJ to produce an effective water cluster interaction with the material to achieve the corresponding maximal depth under different technological conditions.

## 2. Materials and Methods

Austenitic AISI 304 stainless steel was used for the experiments. AISI 304 is widely used in architectural panels, sinks, utensils, sanitaryware, and tubing. It is also used in dairy and food production equipment. The lower thermal conductivity of the material requires coolants and lubricants in sufficient quantity during the machining of AISI 304. The material cannot be hardened by heat treatment processes. The surface can be annealed by heating and rapid cooling to enhance its strength. The chemical and mechanical properties of the AISI 304 used for the experiments are provided in Table 2 and Table 3, respectively.

**Figure 1:**
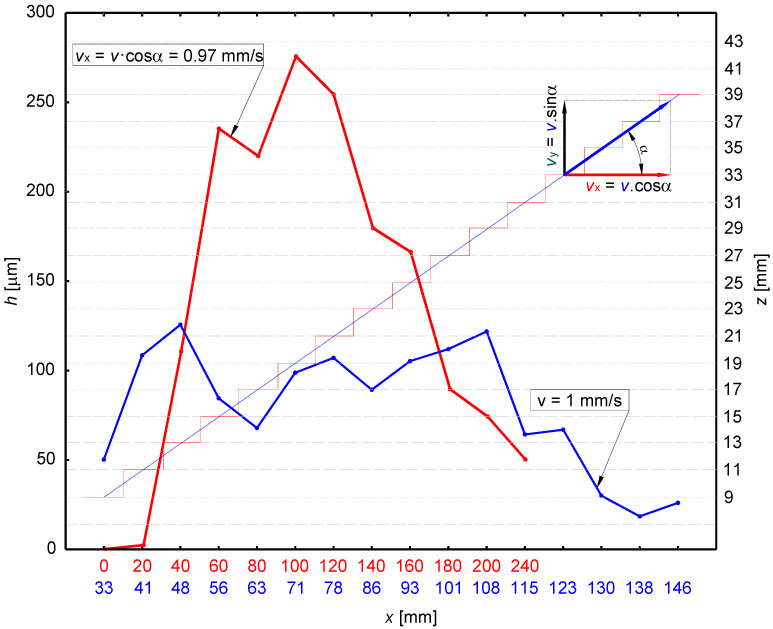
Results comparing the effect of trajectory with constant traverse speed.

The experiments were conducted using the PWJ setup available at the Institute of Geonics v.v.i, Ostrava, Czech Republic. A high-pressure plunger pump (Hammelmann GmBH, Oelde, Germany) with a maximum operating pressure of 160 MPa and a flow rate of 67 L/min was used for the experiments. The required supply pressure (*p* = 70 MPa) was achieved using a bypass flow system and a pneumatic pressure control valve. A Stonage circular nozzle with diameter *d* = 1.19 mm was used to achieve a flow rate of 22.49 L/min at the selected supply pressure. A robotic arm manipulator was used to control the motion and to impart the variable traverse speed of the PWJ head. The pulsations in the system were generated through an ECOSON WJ–UG 630–40 ultrasonic generator with the sonotrode frequency adjusted to *f* = 20 kHz owing to its maximum efficiency. Prior to performing the experiments, a program was loaded into the system describing the motion of the head in the form of a staircase trajectory, as shown in Figure 1 (red line).

From a physical point of view, the standoff distance represents the path travelled by the fluid from the nozzle outlet to the region where it interacts with the material surface. The resulting impact velocity of the jet depends mainly on the friction of the medium in which the fluid flows and the ability to generate pulses. In the process of disintegration or consolidation and removal of residual stresses from the underlying material layers (peening), an inappropriately estimated standoff distance can interfere with the favorable effects of other judiciously selected parameters for a specific material. Residual stresses may either diminish marginally or not have any role to play. Determining the optimal standoff distance is a prerequisite for achieving the desired material characteristics. So far, many experiments have been conducted to clarify the impact of the individual parameters of interaction (Table 1). However, an appropriate method to estimate an appropriate standoff distance has not yet been achieved. Therefore, experiments previously carried out at the optimal standoff distance and the results obtained are somewhat questionable. In previous studies [19,25], standoff distance was determined using the incline trajectory, where the vertical component of velocity (*v_y_* = *v.sinα* mm/s) was neglected. This resulted in the decrement in the actual velocity (*v* = *v_PWJ_* − *v_y_*) of the PWJ, leading to reduction in the impact force. Contrary to previous studies, a new method of trajectory motion is represented by the red color (Figure 1), showing variation in the results, which confirms the hypothesis regarding reduction in the PWJ’s impact force. Comparing the two different approaches, distinct differences can be observed in the vertical and horizontal directions (Figure 1). For example, when the velocity vector is parallel to the solid flat surface at *z* = 19 mm, a depth of more than 250 µm is achieved. Conversely, when the PWJ head is moving on an inclined trajectory with the same vector *v_y_*, the depth is about 100 µm. One can conclude that the previous approach [24] leads to a distortion of the results. Therefore, in the present study, it can be stated that the PWJ head follows a stair trajectory.

**Figure 2:**
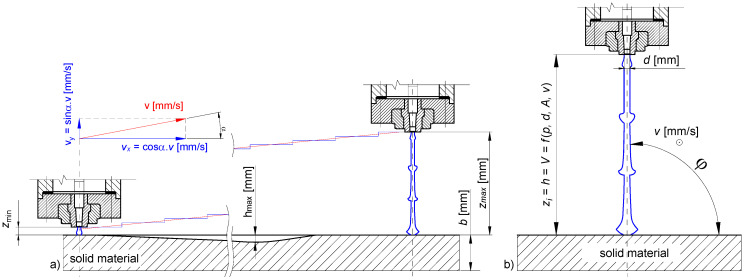
(**a**) Trajectory for the PWJ head; (**b**) PWJ morphology.

The experimental conditions are provided in Table 4. The lowest standoff distance, *z* = 5 mm, was estimated from previous experiments. The nozzle trajectory followed the direct motion of 20 mm with further vertical motion of 2 mm in the standoff distance. The subsequent motion of the nozzle was repeated up to the maximum standoff distance of *z* = 101 mm (Figure 2). A sufficient edge distance was maintained with the traces, with a consecutive distance of 15 mm between each trace to avoid overlapping. The trajectory was repeated for varying traverse speeds ranging from 5 to 35 mm/s.

After performing the experiments, the samples were cleaned and dried using a hot-air dryer to remove any moisture present on the surface for subsequent analysis. The cut samples were subjected to scanning using an optical profilometer (MicroProf FRT with a depth sensor SEN 000 03 having a vertical resolution of 100/30 nm and an accuracy of 1 µm, FRT GmbH, Bergisch Gladbach, Germany). The scanned surface of the trace was then imported into SPIP software (version 6.7.5, Image Metrology A/S, Lyngby, Denmark) for evaluating the erosion depth at each traverse speed. For evaluation of the depth of the traces, each trace was sub-divided into ten equidistant lines and the erosion depth was plotted as the mean and standard deviation of the observed data (OriginPro 8.5). The cross-sections of the samples before imaging were polished using grid paper sizes 300, 600, 1200, 1500, and 2000 using a Saphir 320 polishing machine (ATM Qness GmbH, Mammelzen, Germany). The surface morphology of the selected samples (based on erosion stages) was investigated on a TESCAN MIRA 3 GME scanning electron microscope (SEM, TESCAN ORSAY HOLDING, a.s., Brno, Czech Republic). The SEM images were captured using a secondary electron (SE) detector with an acceleration voltage of 10 kV.

## 3. Results

### 3.1. Erosion Depth

**Figure 3:**
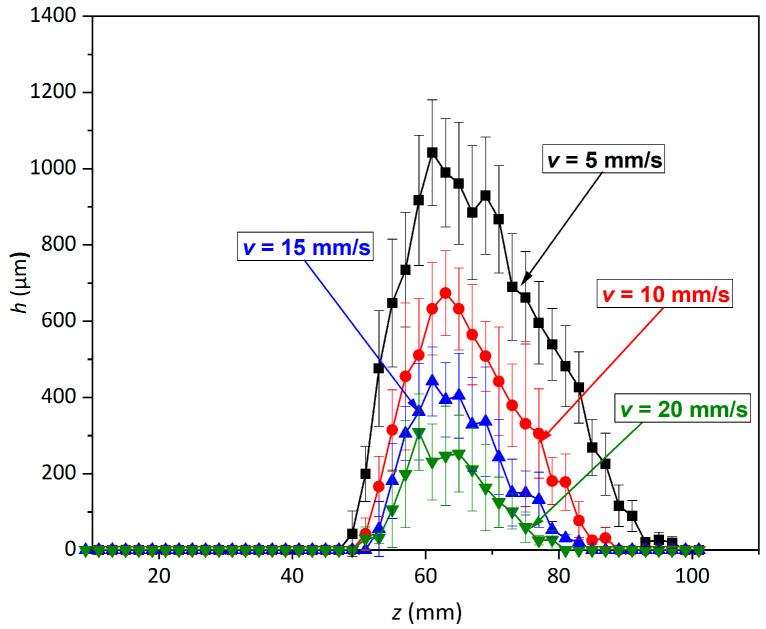
Erosion depth variation with standoff distance *z* (5–101 mm) and nozzle traverse speed *v* (5 (4000 impacts/mm) to 20 mm/s (1000 impacts/mm)).

**Figure 4:**
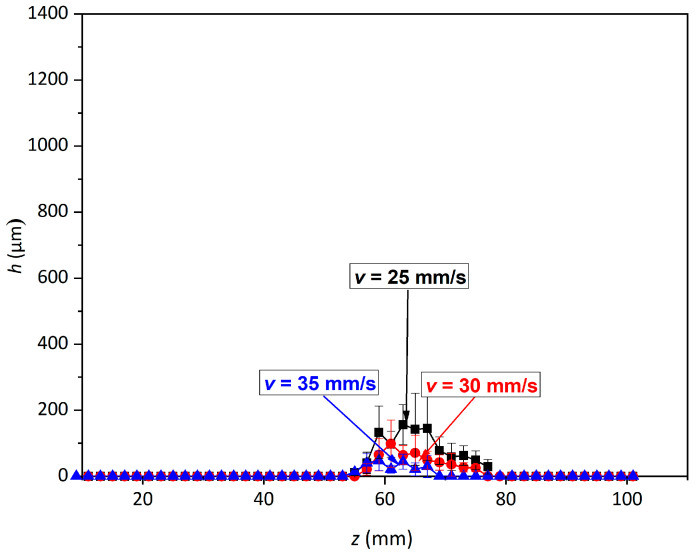
Erosion depth variation with *z* (5–101 mm) and *v* (25 (800 impacts/mm) to 35 mm/s (570 impacts/mm)).

Figure 3 and Figure 4 show the relationship between the depth of erosion and the change in standoff distance (5–101 mm) and with the variation in the nozzle traverse speed (from 5 to 35 mm/s) at a constant pressure of 70 MPa.

Under all traverse speed conditions, the erosion depth showed five distinct regimes of erosion, similar to the findings reported by Hloch et al. [24], where the supply pressure varied from 40 to 100 MPa at a constant traverse speed of 5 mm/s.

**Figure 5:**
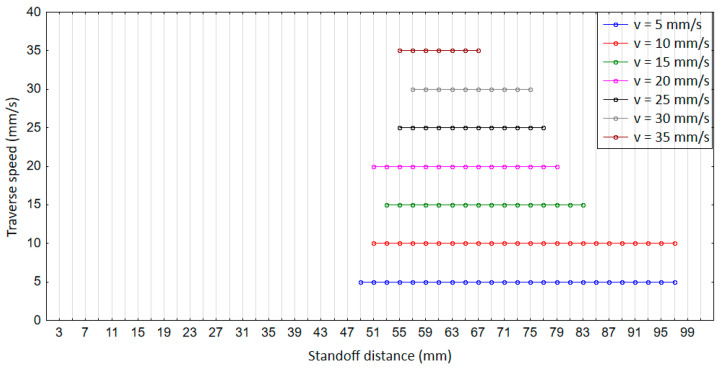
Effect of the PWJ at constant pressure with variation in standoff distance (*z* = 5 to 101 mm) and traverse speed (*v* = 5 to 35 mm/s).

We observed that for all traverse speeds from 5 to 35 mm/s, no measurable erosion depth was obtained before a standoff distance of 47 mm (Figure 5). This occurred due to the non-breakage of the jet at lower *z* (<47 mm), which caused modifications to the sub-surface region without any loss of material. In this range of *z*, the morphology of the PWJ is similar to that of a continuous water jet, which leads to the prevalence of stagnation pressure over impact pressure. This early stage of erosion is known as the incubation regime. For a given nozzle traverse speed (say, *v* = 5 m/s), with further increase in the standoff distance from 47 to 61 mm, the erosion depth accelerates from 42.6 to 1042.2 µm. This is attributed to the jet breaking up into discrete clusters of water droplets impacting the surface periodically. The hydraulic energy of the jet at this range of standoff distance surpasses the ultimate strength of the material (*σ* = 500 MPa), leading to measurable disintegrated grooves. At *z* = 61 mm, the deepest disintegration depth of 1042.2 µm was obtained, which corresponds to the culmination regime of the erosion. The maximum hydraulic energy of the jet is transferred to the material at this standoff distance due to formation of a discrete water front, generating the water hammer phenomenon. With the further increase in *z* from 63 to 97 mm, the energy of the PWJ was attenuated by the aerodynamic drag, resulting in a reduction in the net hydraulic energy transferred to the material. The disintegration depth decreased from 989.8 to 18.9 µm, which is known as the depletion regime of erosion. With the further increase in *z* to >97 mm, the jet dispersed in droplets with a mass lower than the pulses in the prior stages of the PWJ.

Therefore, we concluded that for a given flow rate and traverse speed, the hydraulic energy of the jet induced into the material depends on the standoff distance, corresponding to the morphology of the jet [24]. The total energy of the jet also depends on the number of impacts interacting with the surface. Thus, decreasing the number of impingements decreases the overall impact energy of the PWJ. For *f* = 20 kHz, 20,000 impacts/s are directed to the material. Therefore, with the increase in traverse speed from 5 to 35 mm/s, the number of impacts decreases from 4000 to 570 impacts/mm. However, the energy per impact remains same, which depends on the supply pressure (*p* = 70 MPa) of the jet.

At *v* = 10 mm/s, the incubation period extends until *z* = 49 mm due to the decrease in the number of impacts from 4000 to 2000 impacts/mm. This leads to a decrease in the distribution of the total impact energy along the length of the jet. Therefore, for *v* = 10 mm, the threshold energy induced into the material for overcoming the ultimate strength of the material is reached at *z* = 51 mm. For *z* = 51 to 63 mm, the energy of the jet increases due to the increase in the axial velocity fluctuation associated with the morphology of the jet. This increase leads to deeper disintegrated depth from 42.8 to 673.7 µm for *z* = 51 and 63 mm, respectively. Due to the decrease in the number of impacts per millimeter, the maximum depth (*h* = 673.7 µm) achieved for *v* = 10 mm/s is lower than that for *v* = 5 mm/s (*h* = 1042.2 µm). The further increase in *z* from 63 to 87 mm corresponds to the depletion regime of erosion and the disintegration depth decreases (*h* = 632.4 to 25.3 µm). However, the energy associated with the PWJ at *v* = 10 mm/s is attenuated at an earlier standoff distance (*z* = 63 mm) compared to *z* = 97 mm for *v* = 5 mm/s. This occurs due to the lower number of impacts per millimeter. When *z* > 63 mm, no erosion is observed (termination regime).

Therefore, the decrease in the number of impacts per millimeter (4000 to 570 impacts/mm) with the increment in traverse speed (5 to 35 mm/s) subsequently leads to a decrease in the total hydraulic energy induced into the material and to the convergence of the range of standoff distances for which measurable erosion is observed (*z* = 49 to 97 mm for *v* = 5 mm/s and *z* = 55 to 67 mm for *v* = 35 mm/s). The maximum erosion depth obtained with an increase in the traverse speed decreases due to the lower number of impacts per millimeter (*h* = 1042.2 and 47.3 µm for *z* = 5 and 35 mm/s, respectively). In the previous study, the erosion of AISI 304 [7] by PWJ revealed the existence of three stages with the variation in pressure (10–50 MPa) and standoff distance (40 and 50 mm). Similar stages of erosion were also observed during the erosion of aluminum alloy by PWJ [6]. However, due to the narrow range of parametric values, no particular interaction pattern was observed.

### 3.2. SEM Analysis

**Figure 6:**
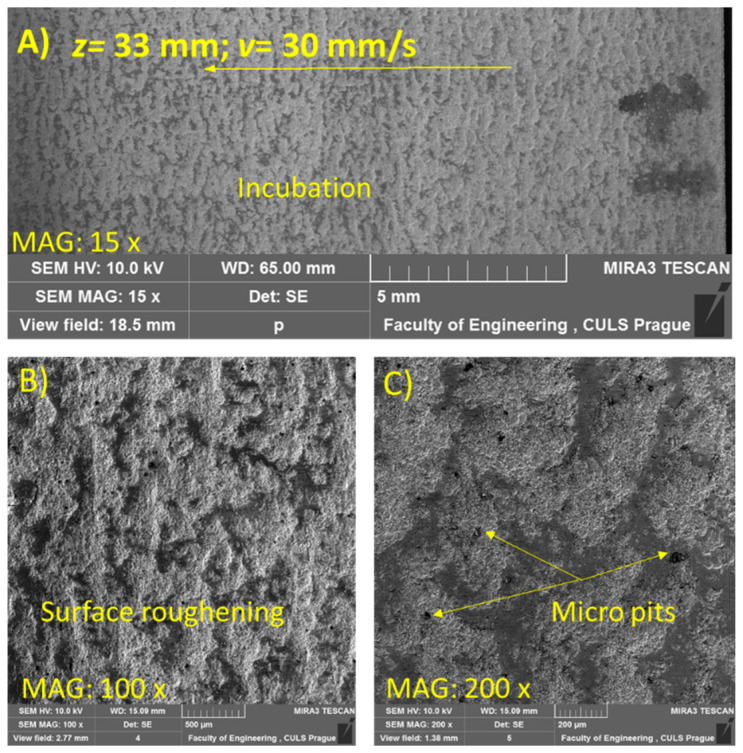
SEM image of sample subjected to *p* = 70 MPa, *z* = 33 mm, and *v* = 30 mm/s (**A**) at magnification 15×, (**B**) At magnification 100× and (**C**) At magnification 200×.

**Figure 7:**
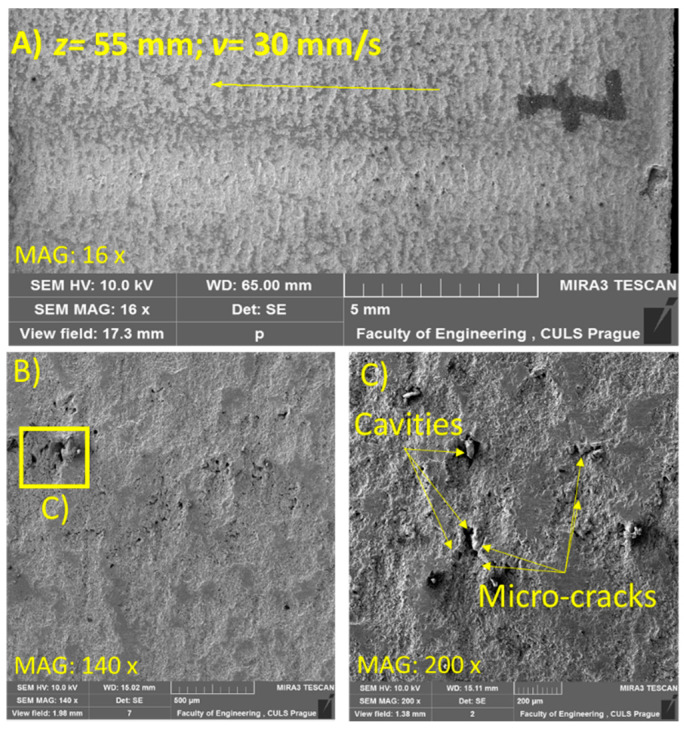
SEM image of sample subjected to *p* = 70 MPa, *z* = 55 mm, and *v* = 30 mm/s (**A**) at magnification 16×, (**B**) At magnification 140× and (**C**) At magnification 200×.

**Figure 8:**
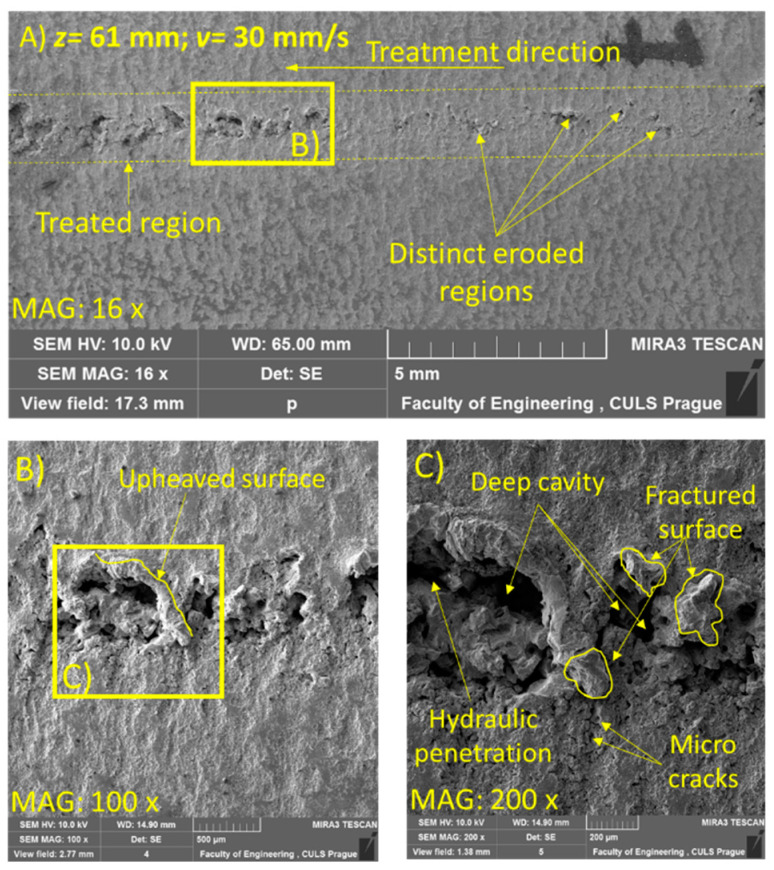
SEM image of sample subjected to *p* = 70 MPa, *z* = 61 mm, and *v* = 30 mm/s (**A**) at magnification 16×, (**B**) At magnification 100× and (**C**) At magnification 200×.

**Figure 9:**
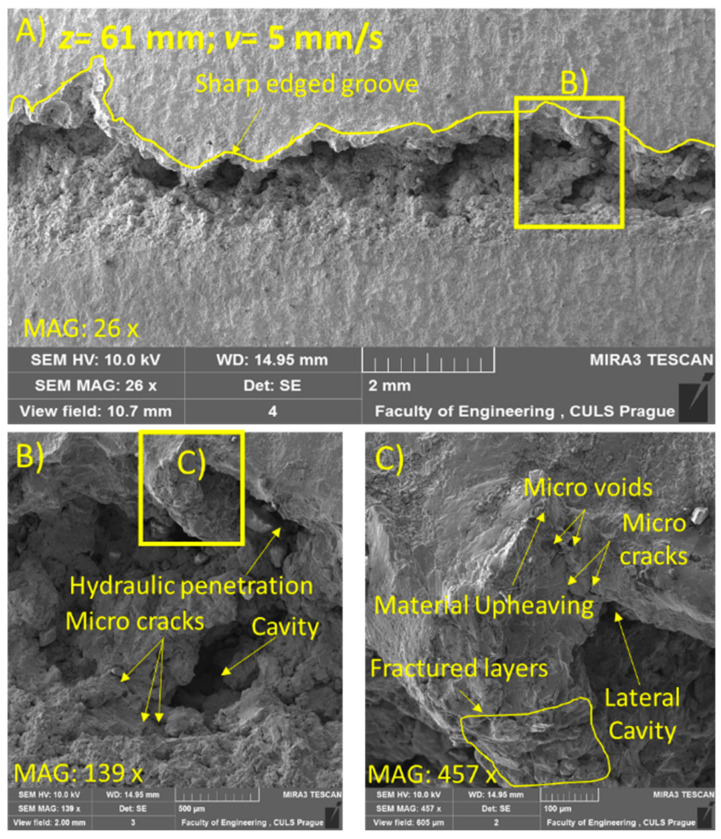
SEM image of sample subjected to *p* = 70 MPa, *z* = 61 mm, and *v* = 5 mm/s (**A**) at magnification 26×, (**B**) At magnification 139× and (**C**) At magnification 457×.

For the SEM analysis, three samples at *z* = 33, 55, and 61 mm were selected due to the visually distinct erosion effects on the samples. Figure 6, Figure 7 and Figure 8 illustrate the morphology of the treated samples at a constant *v* of 30 mm/s, and Figure 9, for *v* = 5 mm/s at *p* = 70 MPa.

The trace shown in Figure 6A describes the incubation stage of the erosion phenomenon where no material loss was observed. The plastic deformation due to the impingement of the jet generates a smooth surface with some micro pits (Figure 6B,C). The effect was observed due to the morphology of the jet, which differed with the change in standoff distance. The standoff distance is an essential factor that describes the jet’s break-up length, allowing it to separate into individual clusters of water droplets. With respect to the specified flow rate, distinct erosion effects were detected due to the difference in the hydraulic energy imparted onto the material. Initially, the breakage of the jet into discrete clusters does not cause any material removal and is approximated as a continuous jet. This is attributed to the hydraulic energy imparted onto the material, which is not sufficient to exceed the material’s ultimate strength (*σ* = 500 MPa).

Figure 7A shows the initiation of the acceleration regime of the erosion, where erosion was observed in the form of cavities (Figure 7B,C). This effect was the consequence of the increased amplitude of velocity fluctuations, which exceeded the threshold limit. With expansion of the standoff distance range from 33 to 55 mm, distinct clusters started to develop [6], which, with periodic impingement on the target surface, impart sufficient hydraulic energy that surpasses the ultimate strength [24] of the material and initiates material removal.

With the further increase in the standoff distance to 61 mm, the generation of an irregular trace with distinct eroded regions was observed (Figure 8A). The erosion features were observed in the form of distinct deep cavities, a fractured surface, and micro-cracks (Figure 8C). This was attributed to the repetitive impingement of the jet, which caused hydraulic penetration through the grains of the material and induced the propagation of compressive stress and shear stress in the material [6]. The induced stresses at this parametric condition led to fatigue failure and caused material removal. The shearing action throughout the lateral dispersion of the jet accounted for the upheaval of the cavity walls (Figure 8B). Micro-cracks occurred due to the propagation of stress waves in the tangential and radial directions.

When the traverse speed was decreased to 5 mm/s, a continuous groove was formed with a sharp edge on one side (Figure 9A). The formation of this non-symmetrical groove was attributed to the action of the reflected waves of the stream. During the impact of the jet, the center of the jet footprint is under compression, while the edges are under the action of tensile stress. This action of tensile stress causes local fractures at the periphery, which, upon subsequent impacts, result in the formation of a non-symmetric groove. In contrast, the trace observed at *v* = 30 mm/s showed irregular eroded regions (Figure 8A). This difference in the eroded trace was attributed to the contact duration of the interacting jet with the target material. On decreasing *v* from 30 to 5 mm/s, the number of impacts increased from 666 to 4000 impacts/mm. This increase in the number of impacts is responsible for the increased hydraulic energy imparted to the surface at the lower traverse speed (*v* = 5 mm/s). The effect was evidenced through the presence of cavities along with features such as micro-cracks, voids, lateral cavities, material upheaving, and fractured layers (Figure 9B,C). The forced shear action caused by the wave propagation within the target material was responsible for the observed surface features. The shear and tensile stress components during the lateral jetting were responsible for the formation of the fractured layers.

**Figure 10:**
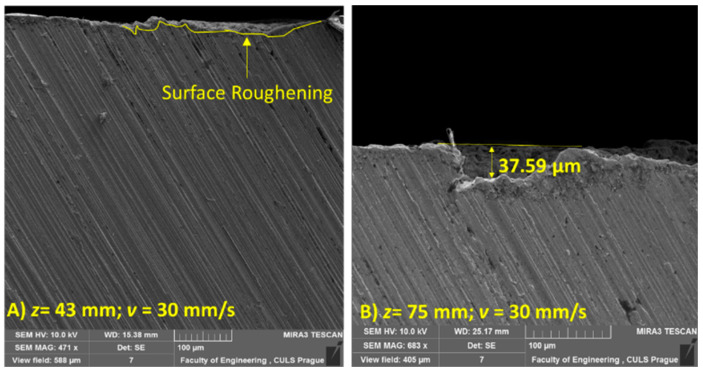
Cross-sectional SEM image of sample subjected to *p* = 70 MPa, *v* = 30 mm/s, and (**A**) *z*= 43 mm or (**B**) *z* = 75 mm.

Figure 10 shows the cross-sectional SEM morphology at *z* = 43 and 75 mm. These cross-sections display different regimes of erosion, i.e., the initial incubation regime (Figure 10A) and the depletion regime (Figure 10B). These stages of erosion are evidenced by surface features such as surface roughening during the incubation stage of erosion due to the prevalence of stagnation pressure over impact pressure. The crater formation of a depth of ~ 37 µm in the depletion stage occurred due to the de-concentrated waves as a result of aerodynamic drag.

From the above morphologies, it was observed that the erosion regimes depend on the total energy imparted to the material, which is a function of standoff distance and traverse speed at a specific flow rate. With the variation in the standoff distance (*z* = 33 to 61 mm), a change in the erosion regimes was observed due to the variation in the jet morphology. A change in the erosion regimes with the variation in traverse speed (from 5 to 30 mm/s) was observed due to the difference in the number of impacts (at *v* = 5 mm/s, 4000 impacts/mm; at *v* = 30 mm/s, 571 impacts/mm). Similar surface erosion features were also observed during the treatment of AISI 304 [16] at standoff distance *z*= 31 mm and varying traverse speeds *v*= 5 to 25 mm/s. At the lower traverse speed, *v*= 5 mm/s, erosion characteristics such as cavities, micro voids and cracks were detected; however, at higher traverse speed, *v*= 25 mm/s, no such characteristics were observed due to the incubation stage.

### 3.3. Comparison of Erosion Interval with Supply Pressure and Traverse Speed

**Figure 11:**
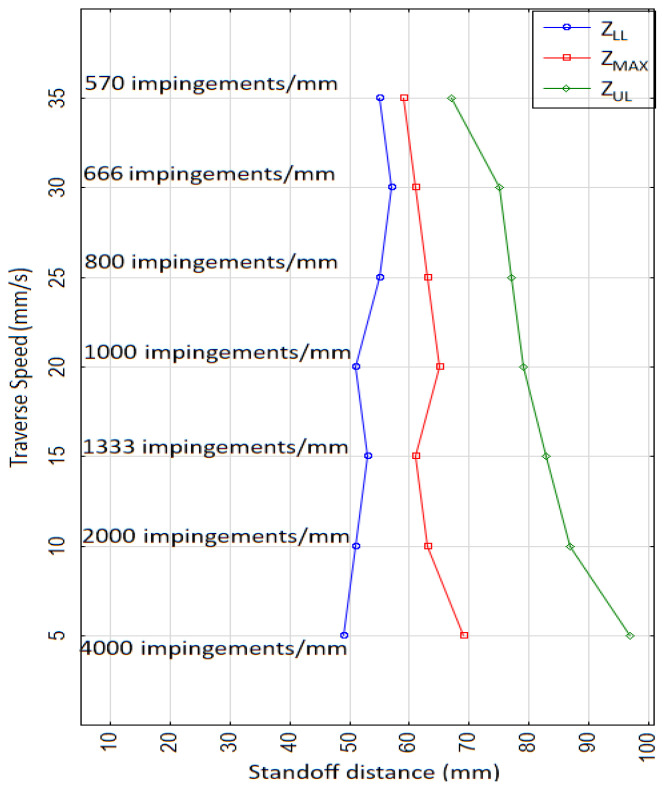
Variation in erosion interval with standoff distance (*z* = 5 to 101 mm) and traverse speed (*v* = 5 to 35 mm/s) at constant pressure (*p* = 70 MPa).

Figure 11 shows the lower limit (*z_LL_*), maximal erosion depth (*z_MAX_*), and upper limit (*z_UL_*) of the erosion interval in terms of standoff distance with varying *v* from 5 to 35 mm/s at a constant *p* of 70 MPa (flow rate *Q* = 22.49 L/min). *z_LL_* is the interface point between the incubation stage and the accelerating stage of erosion, *z_MAX_* is the standoff distance corresponding to the culmination stage of erosion, and *z_UL_* is the interface between the depletion stage and the termination stage of the erosion. We observed that a trapezoidal-shaped erosion envelope was generated in terms of standoff distance with increasing *v* from 5 to 35 mm/s. A broader range between *z_LL_* and *z_UL_* was obtained for *v* = 5 mm/s and a narrower erosion region for *v* = 35 mm/s, which is attributed to decreasing the number of impingements from 4000 to 570 impingements/mm, as explained above. The *z_MAX_* corresponding to different *v* values did not deviate much for *z_MAX_* = 65 ± 5 mm, confirming that the distance required for breaking up the PWJ for a given Q remained the same. However, the energy induced in the material depends on the number of impingements per millimeter corresponding to variations in *z_LL_* and *z_UL_* and the depth values of the disintegration trace.

**Figure 12:**
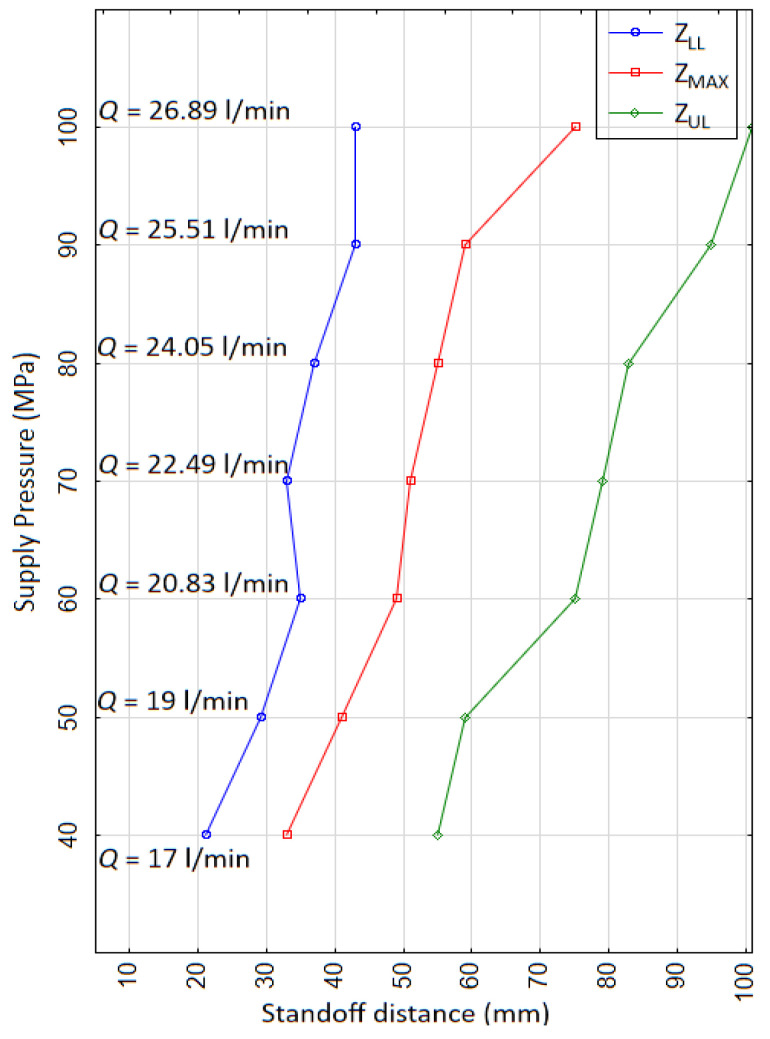
Variation in the erosion interval with standoff distance (*z* = 5 to 101 mm) and supply pressure (*p* = 40 to 100 MPa) at constant traverse speed (*v* = 5 mm/s).

Figure 12 shows the variation in *z_LL_*, *z_MAX_*, and *z_UL_*, representing the erosion envelope with varying *p* of 40 to 100 MPa at a constant *v* of 5 mm/s. The shape of the erosion interval with increasing pressure was that of an inclined parallelogram. The erosion interval range of *z* (*z_UL_* − *z_LL_*) for each pressure remained approximately the same. However, with increasing pressure, the stiffness of the jet increases, requiring a longer distance for breaking up into discrete water clusters. This corresponds to a higher *z_LL_* at higher pressure (43 mm for *p* = 100 MPa) compared to lower pressure (21 mm for *p* = 40 MPa). Due to the similar range of *z* for each pressure, *z_UL_* also shifts toward a higher *z* with higher pressure (55 mm for *p* = 40 MPa, 101 mm for *p* = 100 MPa). The maximum depth corresponding to z_MAX_ also increases with increasing Q due to the increased velocity of the PWJ.

Therefore, the erosion trend with variations in traverse speed and pressure can easily be predicted using both of the above graphs (Figure 11 and Figure 12). The optimal standoff distance to achieve the desired disintegration depth can therefore be determined, resulting in quicker setting of parameters. The lowest level of technological parameters for achieving the same disintegration depth can also be selected using the erosion trends shown in the above graphs (Figure 11 and Figure 12).

## 4. Conclusions

In this paper, we have demonstrated the influence of PWJ on AISI 304 surfaces in terms of erosion effects at varying nozzle traverse speeds (*v* = 5 to 35 mm/s) and standoff distances in the form of a stair trajectory (from *z* = 5 to 101 mm) at a constant pressure of 70 MPa. The conclusions of the analysis are summarized as follows:

- The assessment of the depth profile under each condition showed different erosion phases categorized as incubation, acceleration, culmination, depletion, and termination. The erosion interval depends on the energy distribution as a function of standoff distance and nozzle traverse speed (*z* = 33 to 61 mm at *v* = 5 mm/s and *z* = 55 to 67 mm at *v* = 35 mm/s).
- The maximum erosion depth decreases from 1042 to 47 µm with increasing the nozzle traverse speed from 5 to 35 mm/s due to the number of impingements per unit length of the material (4000 to 570 impacts/mm).
- The surface morphology reveals the presence of erosion characteristics such as micro-cracks, upheaved surface, cavities, and voids due to the repeated impact of the pulsed jet, which allows stress wave propagation (compressive and shear) within the material.
- The erosion interval with the variation in standoff distance and traverse speed (at a constant pressure of 70 MPa) follows a trapezoidal shape, compared to the parallelogram shape obtained by varying pressure and standoff distance (at a constant traverse speed of 5 mm/s). This difference in the erosion geometry is attributed to the variation in the hydraulic energy (pressure variation) and the distribution of constant hydraulic energy (traverse speed variation).

## Figures and Tables

**Table 2:** Composition of AISI 304 [24].

Element	C (wt.%)	Mn (wt.%)	Si (wt.%)	S (wt.%)	Cr (wt.%)	Ni (wt.%)	P (wt.%)
SS (AISI 304)	0.08	2.00	1.0	0.03	18.01	8.22	0.04

**Table 3:** Mechanical properties of AISI 304 [24].

Properties	Brinell Hardness	Poisson Ratio, *v*	Young’s Modulus E (GPa)	Tensile Strength (MPa)	Yield Strength (MPa)	Elongation (%)
Value	88	0.3	193	500	210	45

**Table 4:** Experimental conditions.

S. No.	*f* (kHz)	*p* (MPa)	*d* (mm)	*z* (mm)	*v* (mm/s)	Material	Acoustic Chamber Length *lc* (mm)	Jet Velocity *v_w_* (m/s)	Water flow Rate *Q* (L/min)
1	20.18	70	1.19	5–101	5	Stainless steel AISI 304	22	337.09	22.49
2					10				
3					15				
4					20				
5					25				
6					30				
7					35				

## Data Availability

The data presented in this study are available on request from the corresponding author.
